# Global WaterPack - The development of global surface water over the past 20 years at daily temporal resolution

**DOI:** 10.1038/s41597-024-03328-7

**Published:** 2024-05-09

**Authors:** Igor Klein, Soner Uereyen, Patrick Sogno, André Twele, Andreas Hirner, Claudia Kuenzer

**Affiliations:** 1 Earth Observation Center (EOC), German Aerospace Center (DLR), Weßling, Germany; 2 Institute of Geography and Geology, University Wuerzburg, Wuerzburg, Germany

**Keywords:** Hydrology, Environmental impact

## Abstract

Open surface water across the globe is essential for many life forms and is an important source for human settlements, agriculture, and industry. The presence and variation in time and space is influenced by different natural conditions (e.g. climate, topography, geology) and human use (e.g. irrigation, flood protection). The information on the spatial and temporal distribution of open surface water is fundamental for many disciplines and is also required as an essential parameter for hydrological and climatological modelling. Here, we present a dataset derived from satellite earth observation, which is based on more than 6.3 million single MODIS products with a volume of approx. 300 TB. The resulting dataset reflects the situation of open surface water on a global scale for each day over the time period from 2003 to 2022 at a spatial resolution of 250 m. The dataset enables the analysis of the development of lake and reservoir surface areas, freezing cycles, and inundation areas.

## Background & Summary

Water is essential to all forms of life. The rhythm of continental open surface water forms ecosystems and determines economy and industry^1^. Natural lakes, artificial water reservoirs, wetlands, and floodplains often undergo pronounced seasonal dynamics in their areal extent. The area of open water surface is essentially depending on climate, relief, land cover, and human intervention^1^. These factors can cause regular fluctuations or clear trends over a long period of time or, in extreme weather conditions, cause sudden, large-scale changes^2^. Comprehensive knowledge of global spatial-temporal patterns of water surfaces is of great importance for understanding different natural and man-made processes and their effects^3^. Especially the decrease in freshwater around the world can be detected by satellites^4–6^. Satellite remote sensing has been a key technology to monitor and detect the state of open water surfaces at different temporal and spatial scales^3,7–11^. One of the most important aspects is to detect changes and variations at high temporal resolution. Optical sensors such as Advanced Very High Resolution Radiometer (AVHRR), Moderate Resolution Imaging Spectroradiometer (MODIS), Visible Infrared Imaging Radiometer Suite (VIIRS), Ocean and Land Colour Instrument (OLCI) with medium spatial resolution between 250 and 1000 m provide reasonable compromise between spatial detail, global coverage and daily revisiting time of same region. The MODIS sensors on board of Terra and Aqua satellites were applied for global water mapping at 500 m and daily resolution between 2001 and 2016^12^, at 250 m and 8-day resolution between 2000-2018^13^ and for near real time (NRT) flood mapping^14^. Additionally, VIIRS and MODIS are used to reconstruct water volume of 164 world-wide largest reservoirs at 8-day and monthly temporal resolution^15^. In this context, a detailed recording, observation, and quantification of water surfaces is crucial for accurate modelling and also for the development of effective sustainability strategies, especially in light of ongoing global change^16^.

### Observation of water dynamics with satellite remote sensing

**Fig. 1:**
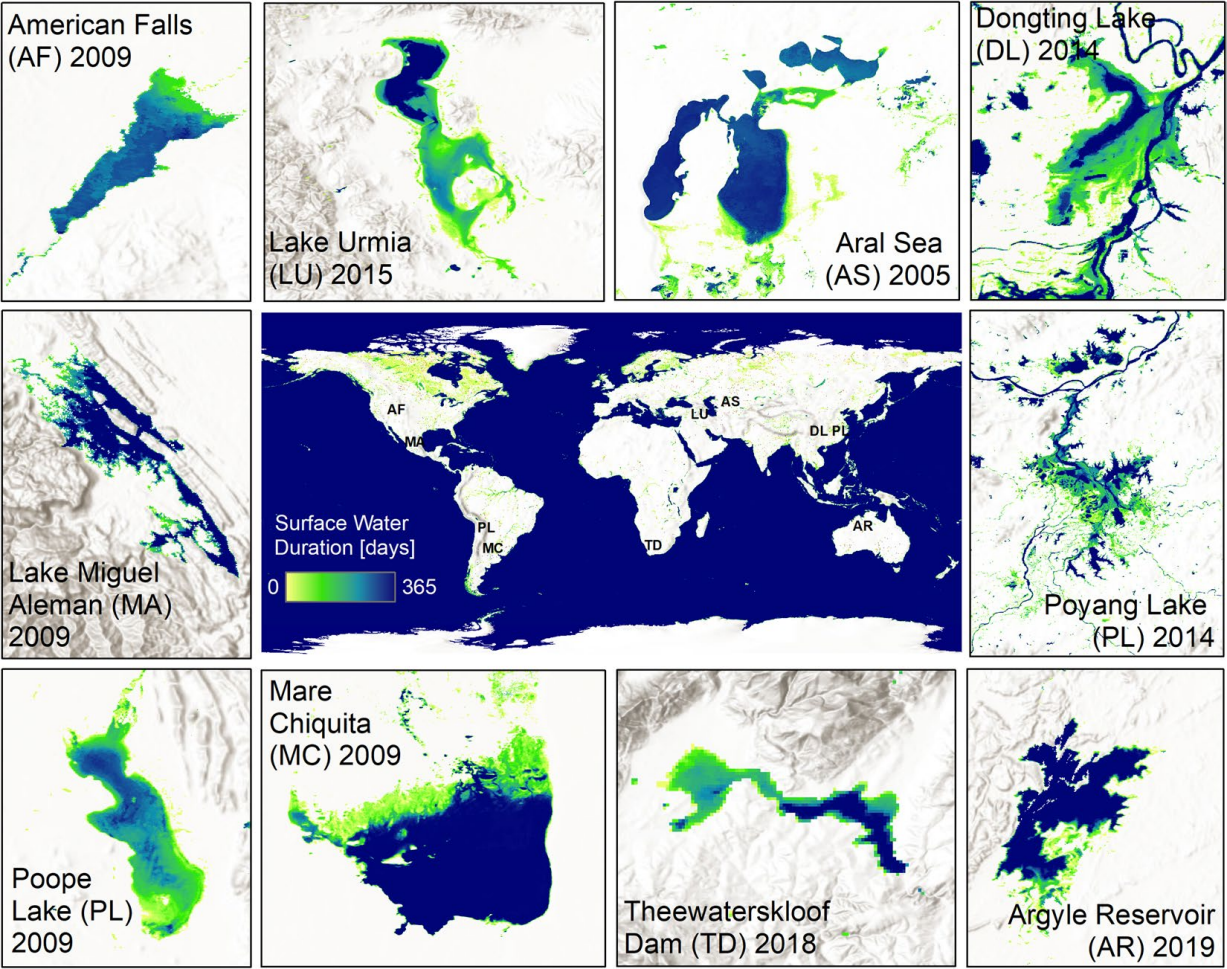
Global WaterPack examples of dynamic water bodies.

The aim of the Global WaterPack (GWP) dataset is the daily worldwide mapping of open surface water. The methodology was published by Klein *et al*.^17^ but the entire range of datasets was not publicly available yet. So far, over 6.3 million individual images from the MODIS sensors between the years 2003 to 2022 have been processed (approx. 300 TB of raw data). MODIS provides daily global coverage which is particularly useful for continental to global scale assessments. As a result of a complex, fully automatic evaluation^17^, the Earth’s surface is divided into two types of binary land cover every day: open surface water and no open surface water. By aggregating the daily results, spatial-temporal patterns of lakes and reservoirs become visible, and their properties can be examined and described. The GWP annual frequency layer shows the number of days within a year on which open surface water occurs in a certain place on Earth (Fig. 1). Depending on the region, drastic differences are visible. While lakes of the mid-latitudes hardly change over the year, the extent of open surface water of lakes and wetlands in the subtropics or in permafrost areas fluctuate widely within a year. Artificial reservoirs also show significant seasonal variations. Lakes in northern latitudes and mountain regions, on the other hand, indicate when and for how long the water freezes because the water mapping methods from optical satellite imagery classify these lakes not as open water. Long-term means, anomalies, and trends can be obtained from the analysis of the derived time series. This enables the observation and quantification of long-term environmental changes.

When considering the entire range of GWP, one can observe the water dynamics as well as lake ice dynamics at a global scale. However, to distinguish lake ice additional datasets on temperature and contextual knowledge are required as this is not flagged in our dataset. Temporal dynamics at different scales, such as seasonal cycles of flood plains along large rivers or the filling and emptying of artificial reservoirs can be clearly determined and investigated^18^. Thus, this dataset provides a comprehensive basis for further quantitative and qualitative evaluations of spatial-temporal water dynamics.

### Improved data for various fields of science and modelling of future developments

The GWP offers great potential for improving global and regional climate and hydrological modelling, water-related risk analysis, or assessing the human impact on various ecosystems. The dataset can also serve as a starting point for long-term worldwide monitoring of historical and future water surface dynamics at high temporal resolution. Furthermore, this enables comparative analyses between different river catchments around the world. Thus, the GWP provides an important input for hydrological analyses, geoscientific modelling, and water resource management. Water availability, water demand, and extreme events such as droughts, heavy precipitation, snowmelt, and floods can be examined with high temporal resolution. Moreover, spatial downscaling to combine high temporal resolution information with higher spatial resolution satellite data such as Landsat, Sentinel-1, and −2 could provide further improvements.

## Methods

**Fig. 2:**
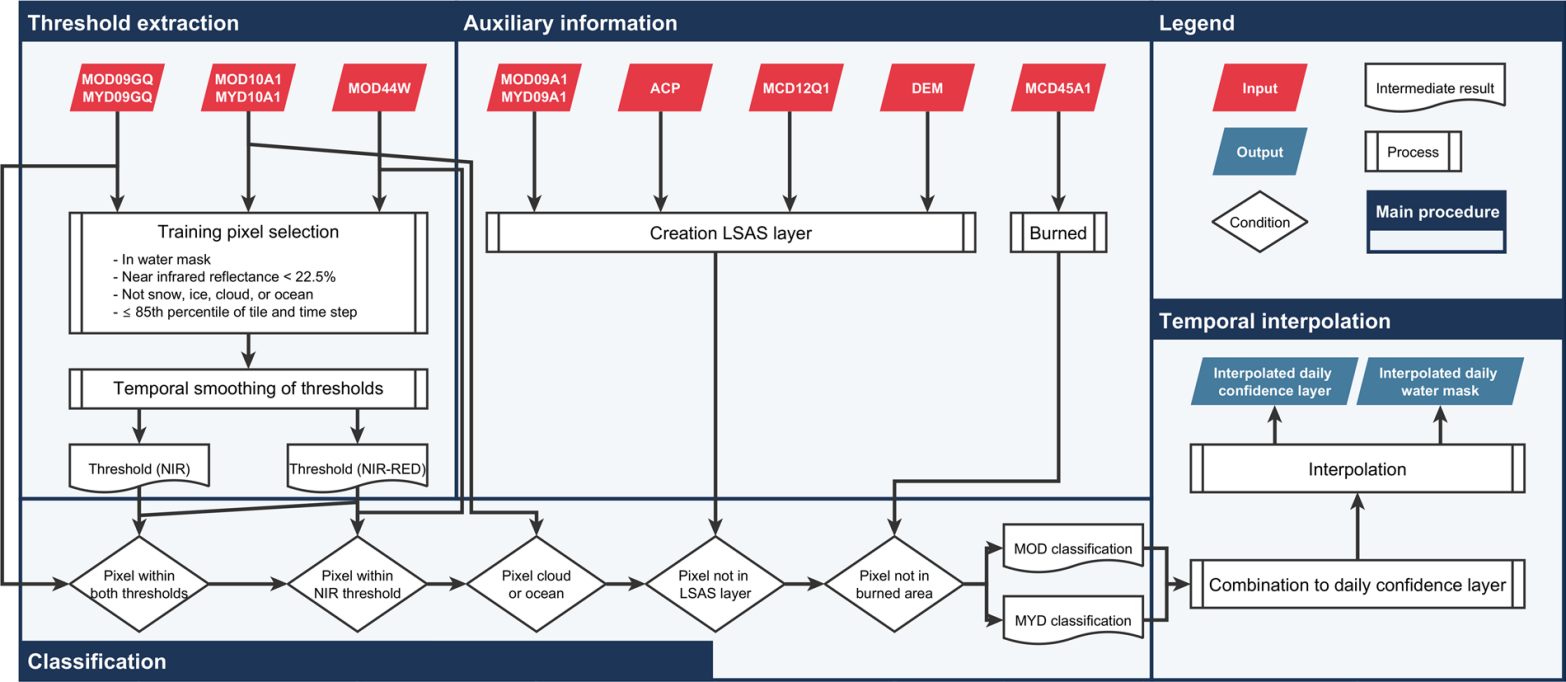
The Global WaterPack workflow is divided into four major processing steps: (I) Training pixel selection and threshold extraction; (II) inclusion of auxiliary information and pre-processing; I(II) dynamic classification of individual images; (IV) temporal interpolation and gap-filling based on closest classification with high confidence.

The GWP was developed to automatically derive open surface water on a global scale from optical MODIS data and exploit the full capacity by combining both daily observations from Terra and Aqua. The dense time series allows for the interpolation of clouds or other data gaps while maintaining high temporal precision. The processing requires a high degree of automatization and consideration of different environmental situations as well as characteristics and quality of used input data products. Figure 2 illustrates the entire processing workflow of GWP which is subdivided into the following five steps: (i) data download and pre-processing, (ii) dynamic training and classification of individual observations, (iii) combination of results from both MODIS sensors, (iv) temporal interpolation and gaps filling based on closest classification with high confidence, (v) post-processing to avoid overestimation of water due to different environmental phenomena. All individual processing steps are explained in the following.

### Data download and pre-processing

The main input datasets for the GWP dataset are daily surface reflectances detected by the MODIS instruments aboard the Aqua and Terra satellites. We utilize the MODIS surface reflectance level-2 gridded products MOD09GQ and MYD09GQ (collection version 6, L2G, https://modis.gsfc.nasa.gov/data/dataprod/mod09.php) containing the near infra-red (NIR) channel (841–876 nm) and the red channel (620–670 nm) at a spatial resolution of approx. 250 m. The MOD09GQ/MYD09GQ products are provided as calibrated spectral radiance values estimating surface spectral reflectance at ground level^19^. Additionally, we use the MODIS daily snow cover gridded level-3 products MOD10A1 and MYD10A1 with a spatial resolution of approx. 500 m (collection version 6, L3G)^20^. These thematic datasets provide a daily estimation of snow cover, cloud cover, and lake ice as well as a static lake and ocean mask^21^. Both used datasets are provided as standardized 10° by 10° tiles (approx. 1,200 × 1,200 km) in Sinusoidal projection. The main global land mass excluding the pole regions (70° to 90°) and remote islands is covered by 206 tiles which results in more than 300,000 input datasets for our process.

### Extracting training pixels and classification of individual observations

We use all pixels which are assigned in the static inland surface water product MOD44W^22^ (https://modis.gsfc.nasa.gov/data/dataprod/mod44w.php) as potential training pixel candidates which are further filtered and reduced based on cloud coverage and spectral characteristics of classified days. The MOD44W is a combination between Shuttle Radar Topography Mission (SRTM) Water Body Data Set (SWBD) and MODIS 16-day composites (before 2009). The layer provides a snap-shot of water areas including transitional environments such as wetlands and estuaries during the operation time of SRTM (February 2000) or derived from MODIS composites. However, as water areas are changing over time, and also can be covered by clouds or lake ice, we have to conduct several conditional tests to guarantee that the extracted training pixels indeed represent surface water at the time of observation. Training pixel candidates which are assigned as cloud, lake ice, or snow in the MOD10A1/MYD10A1 datasets^20^ are excluded. Nevertheless, it cannot be guaranteed that the remaining pixels are free from clouds (especially on cloud fringes) or are not affected by other distortions e.g. due to artefacts resulting from compositing processes, or sun glint. Therefore, further ambiguous training pixel candidates are excluded from the training set by applying a NIR reflectances threshold (thA) to exclude distorted and potential non-water pixels. The remaining pixels are used to calculate dynamic thresholds for the NIR channel and NIR-RED index for actual delineation between water and non-water pixels on a specific day and MODIS tile. These thresholds are calculated as 85^th^ percentiles (thB) of the remained training pixel distributions. Since these daily derived thresholds are highly variable from day to day due to the high variability of cloud coverage and difference in data quality or distortion, we use a temporal 8-day-mean moving window filter to smooth and minimize noise. In this way, the seasonal differences of the spectral response of water are retained, but outliers are reduced. The evaluation process of thA and thB on a global scale is described in detail in a previous study^17^. A pixel is assigned as water when it features values below the dynamic thresholds of both NIR and NIR-RED or if a pixel is characterized by only NIR values below the NIR threshold and additionally assigned as water in the static mask. The additional condition using only NIR threshold and the relation to the static water mask is included to avoid possible omission errors over permanent water bodies due to occasionally data distortions in red band. All pixels which are classified as non-water and are assigned as cloud or ocean within the MOD10A1/MYD10A1^20^ products are labelled as such, remaining pixels are assigned as land (non-open surface water respectively). In this way, we generate a classification for each day, each MODIS tile, and each observation (based on Terra or Aqua) which are used for further processing.

### Combination of results from both MODIS sensors

In this step, we combine both classification results from Terra and Aqua of the same day to create more reliable results and, at the same time, to reduce the overestimation of water related to cloud shadow. In this regard, the combined result can contain the following categories: water detected by both sensors (water-water), land detected by both sensors (land-land), clouds detected by both sensors (cloud-cloud), water-cloud combination, land-cloud combination, and land-water combination. Pixels identified as water or land in both sensors are considered highly confident and are assigned values of 100 for open surface water and 0 for no open surface water. All other categories are considered ambiguous and are assigned a value of 50 to be further processed in the next step.

### Temporal interpolation and gap filling

An important objective is the generation of a gap-free time series. Therefore, ambiguous pixels from the previous step undergo a temporal interpolation and gap-filling process. We utilize the strategy of memory of preceding detection and knowledge of future detection^23^ to interpolate for gaps resulting mostly from clouds or data artefacts. The gap-filling starts with a window of 3 days centered at an ambiguous time step. The sum of all pixel values within the window excluding the centered pixel is divided by the number of pixels within the window minus one. The ambiguous pixel is then replaced by the new value. As long as the centred pixel has a value of 50, the window will increase by an additional time step at the beginning and at the end. Additionally, the classification results are improved by filtering out isolated cases, specifically meaning if only one time-step is classified as water or land. A case is considered an isolated day if a pixel is classified as water (land) but the time steps before and after that are classified as land (water). Such one-time cases are identified in the time series and re-interpreted to the class that has been detected before and after.

After filtering and filling the gaps with the above-described workflow, the output layers are characterized by pixel values between 0 and 100 excluding the value 50. The values can finally be used to create a binary water-no water mask, whereas pixels are re-labelled to land when they show values of less than 50 and to water when they show values higher than 50. In this way, a new cloud and gap-free output is generated. Finally, the number of water classifications within the time series of daily binary masks are counted for each year and each month resulting in annual and monthly open surface water frequency layers which represent the number of days per year and month where one pixel was covered by open surface water.

### Post-processing

To address any overestimation due to topography, building shadows, or dark land surfaces, it is common to use auxiliary datasets^3,24^. For topographic shadows, all slopes steeper than 5° inclination based on a global DEM were included^25^. The classification of urban areas was extracted from the MCD12Q1^26^ product (https://modis.gsfc.nasa.gov/data/dataprod/mod12.php). Additionally, spectrally ambiguous surfaces such as volcanic material were addressed by utilizing the long-term maximum temperature difference between day and night calculated from the ACP dataset^27^. A potential overestimation by burned areas is addressed by calculating the potential maximum surface water area using all available global products^3,12^. A combination of this auxiliary information can be applied to re-classify pixels and reduce overestimation due to ambiguous spectral reflectance. Pixels are re-classified to non-water where both the masks and the temporal profile indicate a high probability of misclassifications due to relief and building shadows, ambiguous land surfaces, and burned areas. Additionally, the static ocean mask from the ESA CCI Land Cover product suite^28^ was used to label ocean pixels within global mosaics because GWP was only processed over main land mass.

### Data quality

Information on the quality of the GWP is provided by means of reliability and observation layers^29,30^. The observation layer is available at a daily temporal scale. This data points out if both MODIS acquisitions of same day were available and had both clear view or not at global scale per pixel and day. The pixel value 0 means no observation and a value of 1 means observation available. Furthermore, we provide reliability layers at a daily, monthly, and annual temporal scale. First, we calculated the reliability layer at daily scale using the number of available observations per pixel within a 30-day interval. In detail, this means 15 days prior to the observation and 15 days after the observation. The sum of available observations (not filled gaps) is then divided by the size of the window. Based on the daily reliability layer, the monthly and annual reliability layers are calculated as the average for corresponding time intervals. The reliability and observation layers only indicate valid values for pixels that have a water pixel in the GWP during the entire investigation period. Furthermore, land pixels are indicated by the value 101 and ocean pixels the value 102.

## Data Records

The Global WaterPack yearly^31^, monthly^32^, daily^33^ datasets are available at DLR’s Geoservice including Web Map Service (WMS) interface for visualization of yearly and monthly datasets and corresponding SpatioTemporal Asset Catalog (STAC) for data search and discovery (links are provided on the DOI landing pages). The file naming convention is:

Daily water map:GWP.OSWF.DAILY.[YYYYMMDD].v1.tif

Monthly water frequency:GWP.OSWF.MONTHLY.[YYYYMM].v1.tif

Annual water frequency:GWP.OSWF.YEARLY.[YYYY].v1.tif

Daily observation:GWP.OSWF.DAILY.[YYYYMMDD].v1.obs.tif

Daily reliability:GWP.OSWF.DAILY.[YYYYMMDD].v1.reliability.tif

Monthly reliability:GWP.OSWF.MONTHLY.[YYYYMM].v1.reliability.tif

Annual reliability:GWP.OSWF.YEARLY.[YYYY].v1.reliability.tif

GWP: Global WaterPack

OSWF: Open Surface Water Frequency

v1: Version 1

**Fig. 3:**
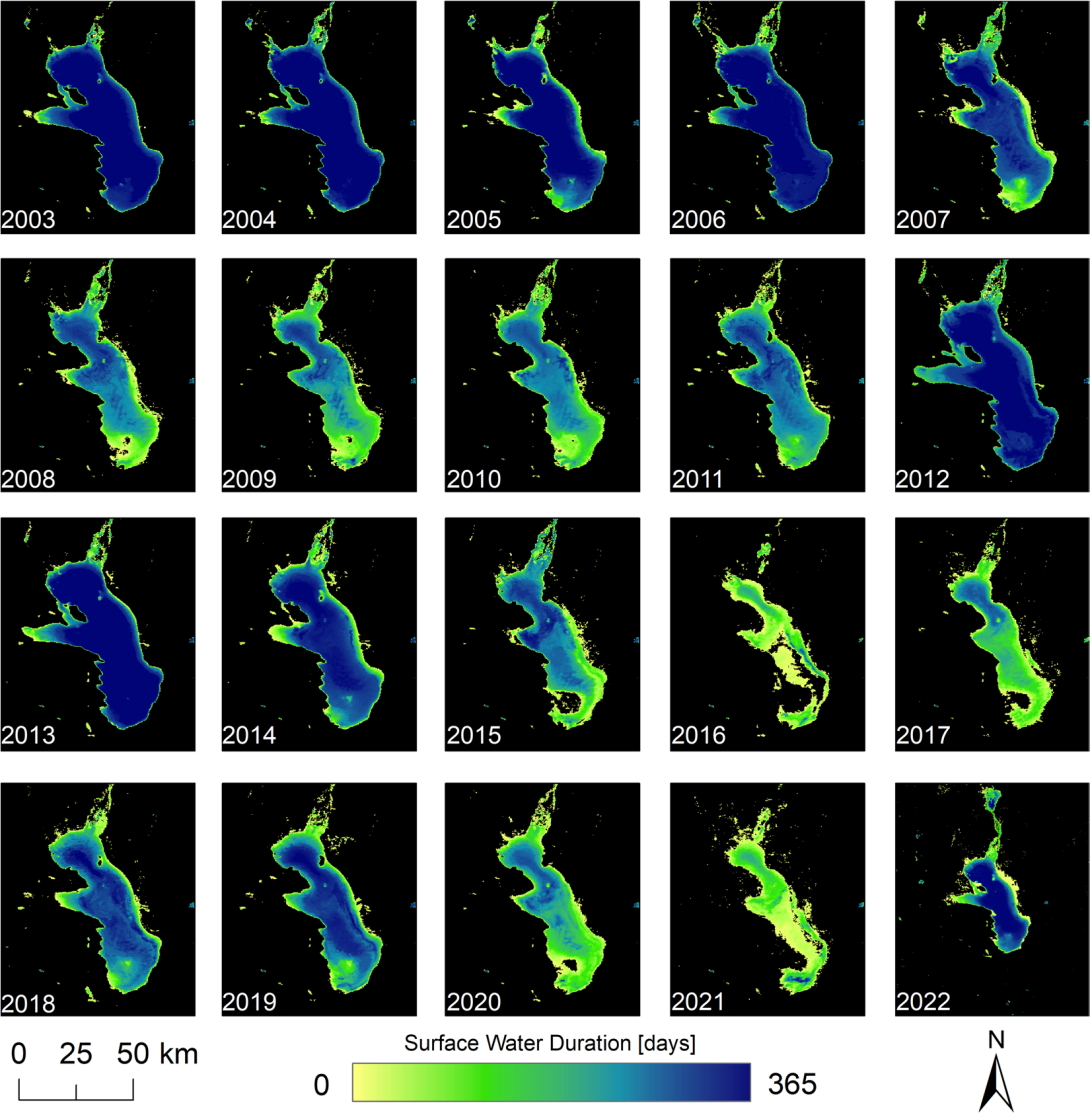
The Global WaterPack annual water frequency of Lake Poopo, Bolivia for the years 2003 until 2022.

**Fig. 4:**
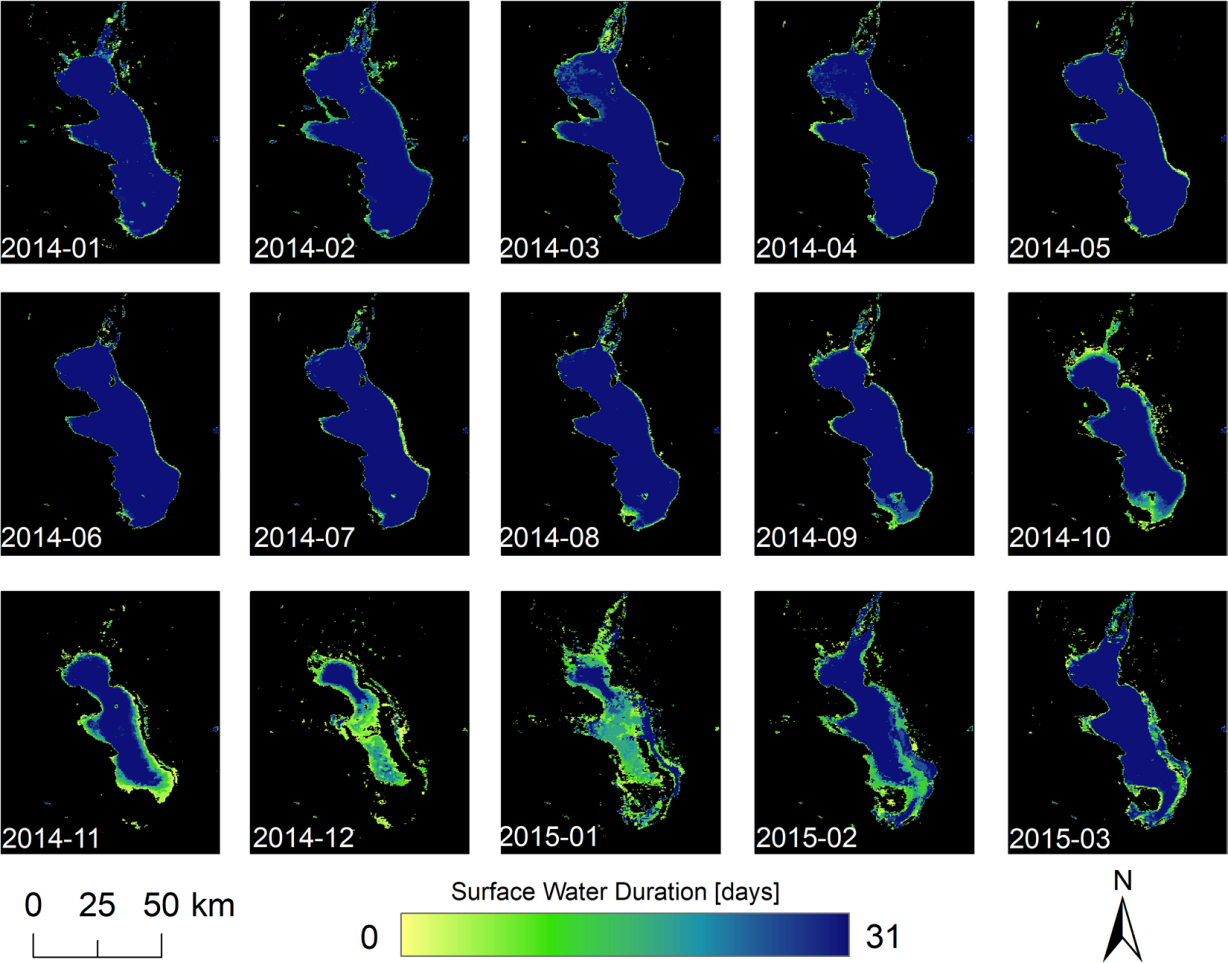
The Global WaterPack monthly water frequency of Lake Poopo, Bolivia for January 2014 until March 2015.

**Fig. 5:**
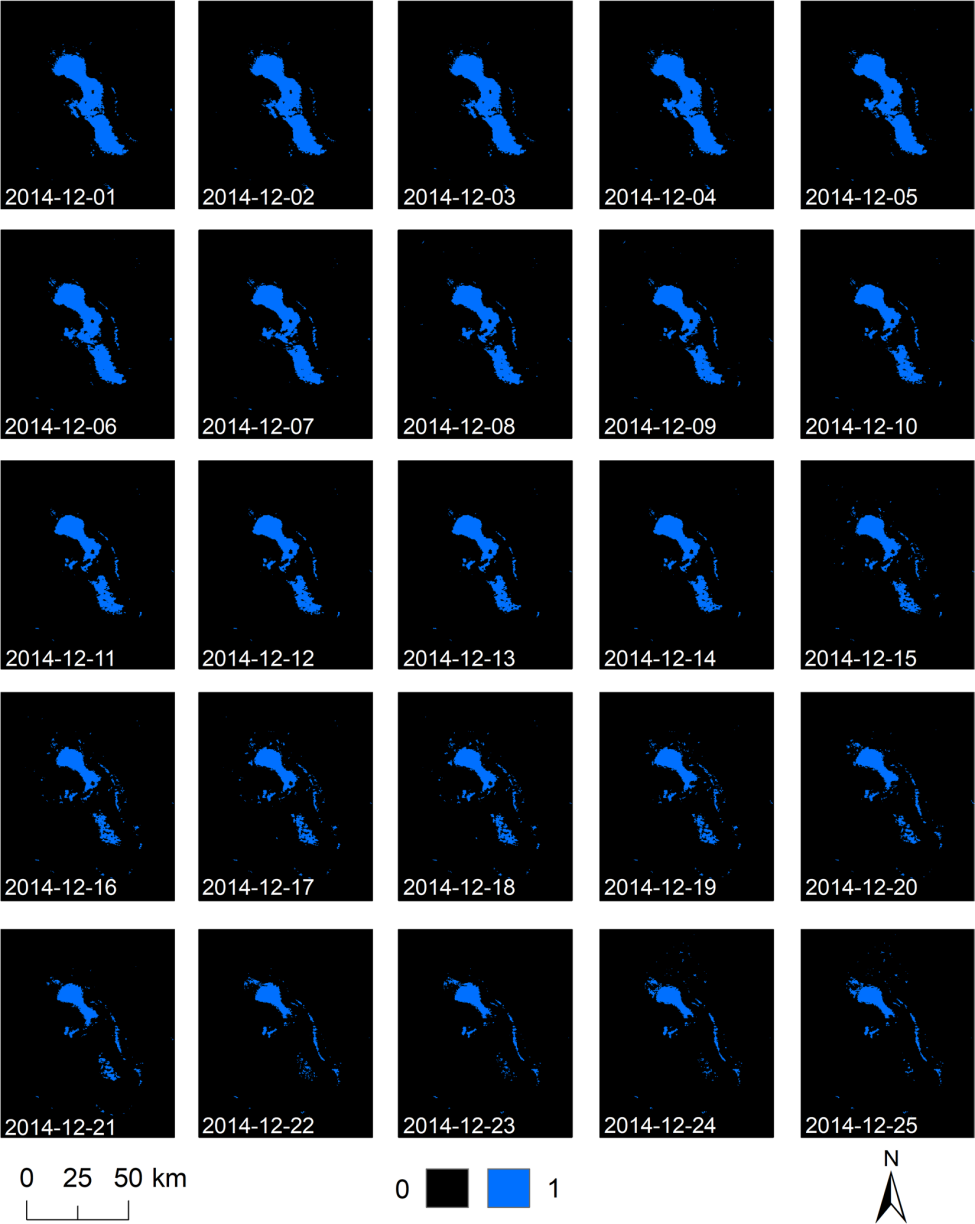
The Global WaterPack daily water masks of Lake Poopo, Bolivia for 1^st^ until 25^th^ December 2014.

All raster files are processed in the Sinusoidal projection inherited from original input MODIS products. For easy handling the final products are mosaicked to one global raster and made available in WGS84 (EPSG:4326). Figures 3–5 demonstrate exemplary yearly, monthly and daily development of Lake Poopo in Bolivia and underline high spatial-temporal variability of surface water.

## Technical validation

The generated daily water masks are validated based on a total of 321 Landsat reference classifications distributed across the globe covering different seasons. The generation of this reference dataset is described in detail in the original method paper^17^.

A random selection of 10,000 water pixels and 10,000 land pixels from the reference dataset was cross compared to the corresponding GWP pixels. For this, the Landsat-based reference data was rescaled and reprojected to 250 m pixels in the MODIS coordinate reference system. Reference pixels that had been included in the definition of the GWP training thresholds (thA, thB) were excluded from validation. Further, water and land validation pixels were filtered to only feature observations with a sub-pixel class fraction of ≥50.

For 250 m pixels with a sub-pixel water fraction of 100%, the overall accuracy was 96.3% with a Kappa coefficient of 93.3. For reference pixels with a sub-pixel water fraction of 75–99.9%, the overall accuracy was 90.1% (Kappa = 79.3). For sub-pixel water fractions of 50–74.9%, an overall accuracy of 58.7% (Kappa = 15.4) could be achieved. The decrease in accuracy with decreasing sub-pixel water fractions is due to mixed pixel effects occurring at the medium spatial resolution of 250 m. Thus, especially narrow rivers, channels, and very small water bodies are underestimated which is mirrored in an elevated omission error of 72.4% for low sub-pixel fractions of water (50–74.9%). A summary of this quantitative accuracy assessment is given in Table 1. In regards to accuracy, the user has to keep in mind that presented validation approach and usage of Landsat represents the agreement between automatic mapping derived from MODIS sensor and manually digitalized water areas from higher spatial resolution Landsat data at 30 m. Even though, such cross comparisons are common, the used digitalized water area are a proxy for ground truth and the provided measures demonstrate the agreement between the water maps derived from two satellite sensors with different spatial and temporal resolutions.

**Table 1:** Global accuracy assessment of GWP using Landsat-based reference data sub-pixel water fractions.

	Sub-pixel water fraction in rescaled, reprojected reference dataset		
	100%	<100% - 75%	<75% - 50%
Omission error	7.8%	20.7%	72.2%
Commission error	0.5%	0.9%	13.1%
Water mapping accuracy	91.7%	78.5%	23.1%
Overall accuracy	96.3%	90.1%	58.7%
Kappa coefficient	93.3%	79.3%	15.4%
F-score	95.4%	86.6%	35.6%

## Usage Notes

GWP datasets are updated yearly, however the dataset version described and peer reviewed in this manuscript relates to data collected from 2003–2022. The GWP dataset was developed to provide scientists and modellers with daily and continuous information over 20 years on global open surface water without requiring time-consuming manual delineations.

Even though for many regions reasonable accuracies are achieved, the user has to consider some limitations. Especially water bodies with a high ratio of mixed-pixels to total area as well as water bodies in tropical or high latitude regions with persistent and long-lasting cloud cover should be interpreted with care. In regions with long-lasting cloud cover or polar night the majority of daily results are interpolated. The time span between clear twice-daily MODIS data can be several months which can lead to biased results due to data gap filling. Furthermore, open surface water covered by vegetation or high sediment load can be classified as non-water due to its ambiguous reflectance in NIR and red spectrum. Water bodies which experience freezing and are covered by ice and snow are classified as no open surface water for these periods. Therefore, lake ice phenology is indirectly contained in the dataset which can be extracted by user using e.g. temperature data or contextual knowledge. On the other hand, additional underestimation of open surface water can occur due to increased fraction of mixed pixels during freezing and thawing processes.

## Data Availability

The generation of the GWP is conducted using multiple processing steps including data acquisition and preparation, classification, interpolation, as well as enhancement of overestimated pixels. Global MODIS data was downloaded and stored in computing environments of DLR’s Earth Observation Center. Further processing tasks have been performed in internal CPU and GPU clusters available at DLR’s Earth Observation Center using DLR proprietary software along with specialized Python (v3.8, https://www.python.org/downloads/windows/) and IDL (v8.0, https://www.nv5geospatialsoftware.com/Products/IDL) scripts. Due to the utilization of proprietary tools, it is not possible to openly disclose the implemented processing pipeline to the public. The calculation of the global mosaics at different temporal scales which are available for download at the Geoservice of the DLR Earth Observation Center were carried out using GDAL (Geospatial Data Abstraction Library v.3.6, https://gdal.org/index.html). The corresponding scripts are available at https://download.geoservice.dlr.de/GWP/files/code/.
